# Supplementary material for: Metallic Nanoparticle Integrated Ternary Polymer Blend of PVA/Starch/Glycerol: A Promising Antimicrobial Food Packaging Material
Source: Polymers (Basel). 2022 Mar 29;14(7):1379. doi: 10.3390/polym14071379 (PMC9002704; doi:10.3390/polym14071379)
Supplement: Supplementary file 1 [file polymers-14-01379-s001.zip › Table S1.pdf]

Table S1: Minimal inhibitory and bactericidal concentration of Copper oxide and Zinc oxide nanoparticles against human pathogens

	Tetracycline		CuO		ZnO	
	MIC (µg/ml)	MBC (µg/ml)	MIC (µg/ml)	MBC (µg/ml)	MIC (µg/ml)	MBC (µg/ml)
Klebsiella pneumoniae	100	100	5	5	5	7.5
S aureus	0.78	1.56	5	5	5	5
