# Supplementary figures and images for: Metallic Nanoparticle Integrated Ternary Polymer Blend of PVA/Starch/Glycerol: A Promising Antimicrobial Food Packaging Material
Source: Polymers (Basel). 2022 Mar 29;14(7):1379. doi: 10.3390/polym14071379 (PMC9002704; doi:10.3390/polym14071379)

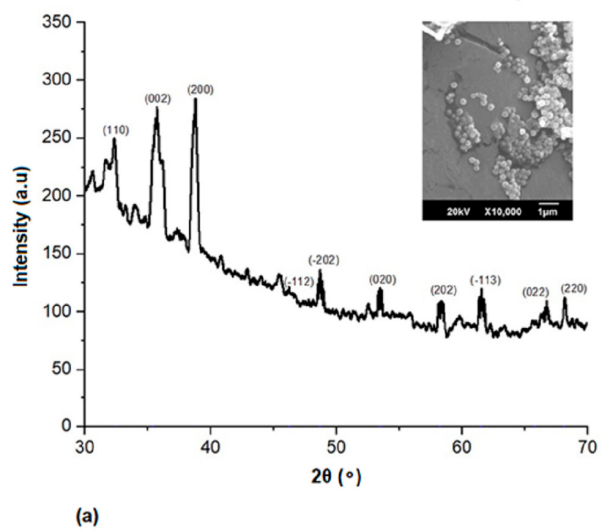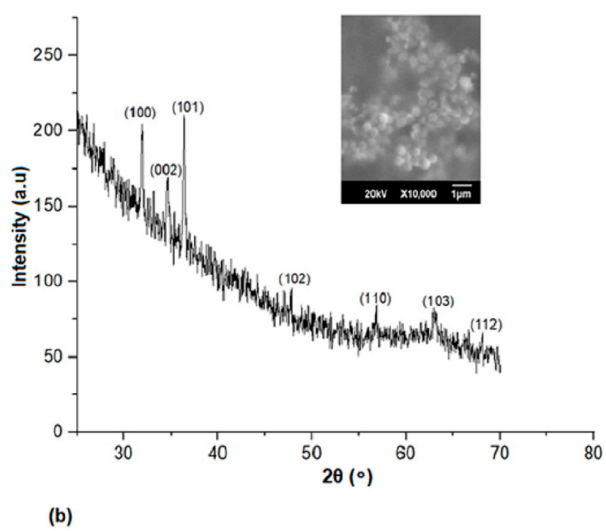

Supplement: Supplementary file 1 [file polymers-14-01379-s001.zip › Figure S1.pdf]

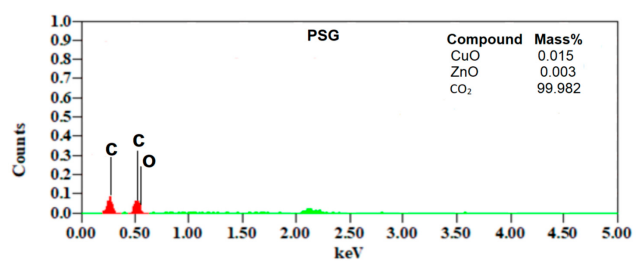

(a)

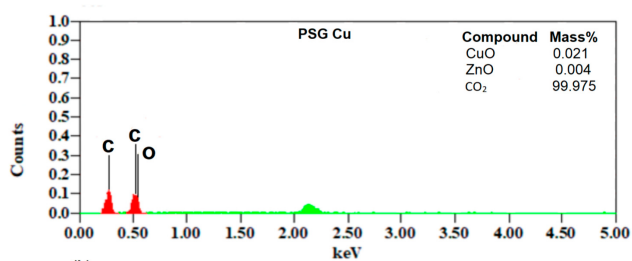

(b)

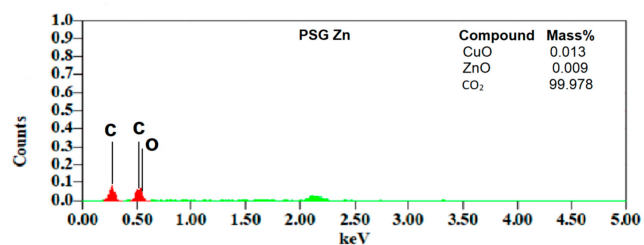

(c)

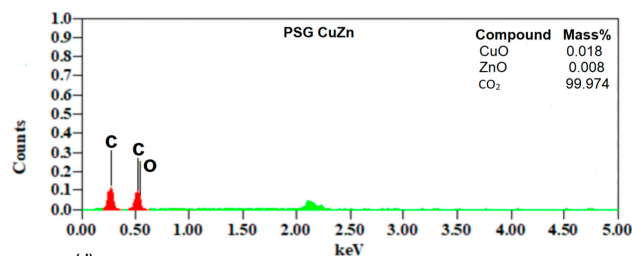

(d)

Supplement: Supplementary file 1 [file polymers-14-01379-s001.zip › Figure S2.pdf]
